# CAR-engineered T cell therapy as an emerging strategy for treating autoimmune diseases

**DOI:** 10.3389/fmed.2024.1447147

**Published:** 2024-10-10

**Authors:** Jovana Vukovic, Dzihan Abazovic, Dusan Vucetic, Sanja Medenica

**Affiliations:** ^1^Institute for the Application of Nuclear Energy - INEP, University of Belgrade, Belgrade, Serbia; ^2^Faculty of Medicine, University of Belgrade, Belgrade, Serbia; ^3^Institute for Transfusiology and Hemobiology, Military Medical Academy, Belgrade, Serbia; ^4^Faculty of Medicine, University of Montenegro, Podgorica, Montenegro; ^5^Department of Endocrinology, Internal Medicine Clinic, Clinical Center of Montenegro, Podgorica, Montenegro

**Keywords:** autoimmunity, CAR-T, CAAR-T, CAR-Treg, immunotherapy, cell therapy, regenerative medicine

## Abstract

CAR-T therapy has demonstrated great success in treating hematological malignancies, which has led to further research into its potential in treating other diseases. Autoimmune diseases have great potential to be treated with this therapy due to the possibility of specific targeting of pathological immune cells and cells that produce autoantibodies, which could lead to permanent healing and restoration of immunological tolerance. Several approaches are currently under investigation, including targeting and depleting B cells via CD19 in the early stages of the disease, simultaneously targeting B cells and memory plasma cells in later stages and refractory states, as well as targeting specific autoantigens through the chimeric autoantibody receptor (CAAR). Additionally, CAR-engineered T regulatory cells can be modified to specifically target the autoimmune niche and modulate the pathological immune response. The encouraging results from preclinical studies have led to the first successful use of CAR-T therapy in humans to treat autoimmunity. This paved the way for further clinical studies, aiming to evaluate the long-term safety and efficacy of these therapies, potentially revolutionizing clinical use.

## 1 Introduction

Autoimmune diseases encompass a heterogeneous group of conditions that can be manifested in a particular organ or at the systemic level. Despite their diversity, they share common immunopathogenic mechanisms involving dysregulated T and B cell responses. A combination of factors such as genetic predisposition and environmental triggers mainly influences the development of autoimmune disorders, leading to the breakdown of self-tolerance and pathological immune reactions, causing tissue destruction (1). T and B lymphocytes play crucial roles in the immune system, acting as the main drivers of adaptive immunity. CD4+ T lymphocytes orchestrate immune responses by releasing cytokines and activating other immune cells (2). When CD4+ T lymphocytes malfunction, autoreactive T cells can be generated, leading to persistent inflammation and tissue damage. Similarly, when B lymphocytes are not appropriately regulated, they can start producing autoantibodies that target self-antigens, contributing to tissue damage (1, 3, 4). An in-depth understanding of these processes is essential for developing precisely targeted interventions without damaging healthy tissue.

Ensuring the desired immune response is crucial to re-establishing immune tolerance and effectively mitigating the consequences of the disease. Conventional treatments for these diseases tend to focus on symptom management, often resulting in undesirable side effects and limited effectiveness. However, a more effective strategy would involve treatments that focus on the root cause of the disease, such as chimeric antigen receptor T (CAR-T) cell therapy. Recent advancements in CAR-T therapy hold promise in treating autoimmune diseases.

**Figure 1:**
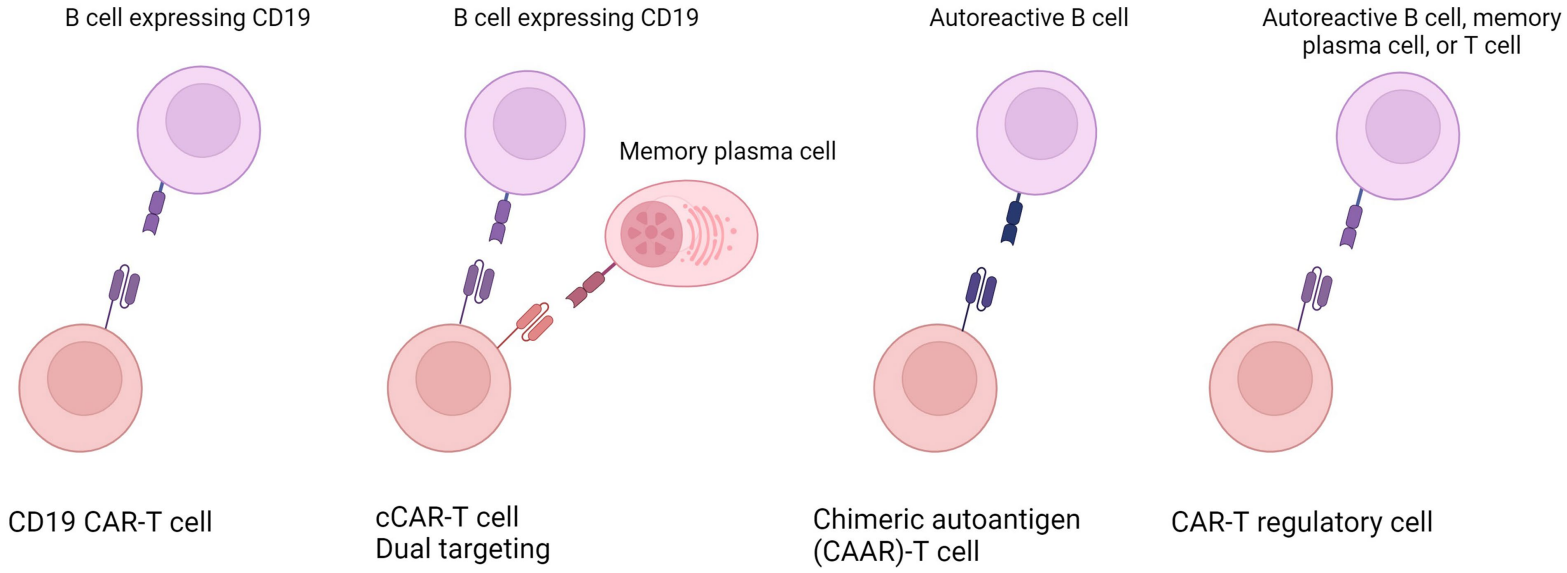
Overview of the various strategies employed in engineering CAR-T cells to tackle autoimmune diseases. These innovative approaches encompass (from left to right) universal targeting of all B cells; the dual targeting or compound CAR engineering targeting B cells and additional cells such as memory plasma cells; restricted B cell depletion or specific targeting of only autoreactive B cells; and the engineering of T regulatory cells. Created in Biorender.

CAR-T therapy involves genetically engineering a patient’s T cells to express chimeric antigen receptors (CARs) that target specific antigens. CD19 is the most investigated target for CAR-based therapy. It is expressed in normal and neoplastic B cells and maintained at a high level during all stages of B-cell development (5, 6). CD19+ malignancies were the first cancers to be eliminated by CAR-engineered human T cells administered intravenously to mice (7). Various CD19 CARs successfully eliminated B cell tumors, resulting in ongoing clinical trials and FDA approval (8, 9). Similarly, B-cell depletion could also be a promising therapeutic strategy for treating autoimmune diseases. Moreover, other strategies are under investigation, such as restricted B cell depletion by targeting autoantigens, dual targeting, and engineering regulatory T cells (Tregs), which will be further reviewed (Figure 1).

### 1.1 Chimeric antigen receptor characteristics, design, and manufacturing

Chimeric antigen receptors (CARs) are modified receptors that alter the specificity and activity of T lymphocytes and other immune cells, by bringing together the variable regions of high affinity monoclonal antibodies with intracellular signaling components of the T-cell receptor (TCR) complex. Their modulatory structure consists of four domains: ligand-binding, spacer, transmembrane, and cytoplasmic domains (10). CARs bind to antigens through an extracellular portion composed of a ligand-binding domain and spacer, typically constructed using single-chain variable fragments (scFv) derived from antibodies, Fab fragments from libraries, or natural ligands. Most commonly used, scFvs can function autonomously or as modular units for CAR-T cell therapies, determining the ability of modified T cells to recognize and target desired antigens (11). CAR-mediated recognition is MHC-independent, allowing it to overcome tolerance to self-antigens and target any chosen antigen expressed on the cell surface (12, 13). CARs also control T cell growth and persistence, impacting both efficacy and safety. Associating CARs with costimulatory ligands, chimeric costimulatory receptors, or cytokines can improve T cell efficacy, specificity, and safety (11, 14, 15).

T cells that express first-generation CARs lacking a co-stimulatory domain are insufficient for T cell activation and show limited *in vivo* effectiveness (16, 17). The second-generation CARs were developed to address this issue by adding a co-stimulatory domain, commonly CD28 and 4-1BB (CD137). Co-stimulatory domains provide additional signals upon antigen recognition, which is crucial for enhanced proliferation, cytokine release, cytotoxic activity, memory formation, and persistence of T cells (18). CAR-T cells with the CD28 intracellular co-stimulatory domain demonstrated significantly increased proliferation and persistence compared to those without a co-stimulatory domain (19). Moreover, CAR-T cells containing the CD28 domain showed better early growth and cytotoxic activity than those containing the 41BB costimulatory domain, which showed better long-term survival (20). Following, the third-generation of CARs incorporates multiple co-stimulatory domains, while fourth-generation CARs are engineered to express additional inducible transgenic elements, typically for inducible cytokine secretion, to improve T cell function and reduce off-target toxicity (14, 21, 22).

The process of CAR-T cell manufacturing begins with harvesting specific T cell subsets from patients via leukapheresis, washing them, and enriching them for specific subsets. Subsequently, T cells undergo activation to ensure adequate transduction of CAR cDNA. CAR gene delivery relies on viral and non-viral gene transfer systems. Following transduction, modified T cells are expanded and, after final concentration, ready to give back to patients (23).

## 2 CD19-targeted CAR-T cell therapy for autoimmune diseases

Systemic lupus erythematosus (SLE) is a systemic autoimmune disease affecting various organs and tissues. A key role in SLE immunopathogenesis play B cells by producing autoantibodies targeting double-stranded DNA, that cause inflammation and tissue damage. Despite several therapeutic approaches targeting autoreactive B lymphocytes, SLE remains incurable (6). Due to sustained expression of CD19 in B-cell lineage, CD19 CAR-T therapy can potentially treat lupus by offering precision targeting and regulating the overall immune response (5, 6). Modifying T cells with CD19-targeting CAR and targeting CD19+ B cells may restore immune balance, decrease autoantibody production, and reduce the inflammatory response associated with lupus. Preclinical results indicate that anti-CD19 CAR-T cells are effective in depleting CD19+ B cells in a lupus model, eliminating autoantibody production, reversing disease manifestation in target organs, and increasing lifespan, suggesting long-term efficacy of this therapeutic approach (24, 25). Recently, encouraging effects have been shown in humans for anti-CD19 CAR-T treatment of SLE. A patient with severe SLE was treated with autologous CD19 CAR-T cells and exhibited complete and sustained depletion of circulating B cells, followed by the disappearance of dsDNA autoantibodies. The therapy showed no adverse events and suggested wider application in treating severe autoimmune (26). This result was confirmed by another study that treated five refractory SLE patients with anti-CD19 CAR-T. All patients demonstrated sustained remission 1 year following the treatment. Three months following the treatment, naïve B cells reappeared without a relapse of SLE symptoms. This finding suggests that CAR-T in autoimmunity, due to different microenvironments, demonstrates transient persistence and achieves acute B cell aplasia, effectively resetting the immune system (27). In a recent update to its SLE management guidelines, the European League Against Rheumatism (EULAR) included CAR-T as one of the treatment options for patients with refractory SLE (28). However, further clinical trials are needed to evaluate the long-term safety and efficacy of anti-CD19 CAR-T in SLE.

Furthermore, another successful human treatment with anti-CD19 CAR-T therapy has been reported for treating another autoimmune disease, myasthenia gravis, caused by a B-cell-driven dysfunction of neuromuscular transmission, often mediated by anti-acetylcholine receptor (anti-AchR) antibodies. A patient with refractory, anti-AchR positive generalized myasthenia gravis was successfully treated with anti-CD19 CAR-T therapy, followed by reduced anti-Achr antibodies without any CAR-T therapy-related side effects, demonstrating improved muscle strength and fatigue (29).

Additionally, two separate studies reported successfully treating two patients suffering from refractory antisynthetase syndrome with anti-CD19 CAR-T therapy (30, 31). This syndrome is characterized by the presence of autoantibodies directed against aminoacyl transfer RNA synthetase, along with clinical features such as interstitial lung disease, myositis, and arthritis. The therapy completely eliminated circulating B cells as well as pathogenic autoantibodies, and both patients experienced improved disease-related symptoms. One patient had a transient increase in myalgia and creatinine kinase concentration due to one of the most common side effects of CAR-T therapy, cytokine release syndrome (CRS), which resolved within 3 days (30). The second patient also experienced mild CRS, which might have stimulated preexisting autoreactive T cells, as an increased number of CD8+ Temra cells in peripheral blood were found, accompanied by exacerbations of muscle pain a few days following the treatment. Treatment with azathioprine and mycophenolate mofetil normalized Temra subsets and potentially contributed to the patient’s beneficial outcome (31).

## 3 Dual targeting for optimal treatment of autoimmune diseases

Early in the disease course, CD19-based therapy may prevent autoreactive plasma cell accumulation, but later, memory plasma cells may accumulate and lead to persistent autoantibody production despite B-cell depletion (32, 33). CD19, one of the earliest and most specific markers of B-lineage cells, may not be expressed in all plasma cells. Plasma cells express CD19 heterogeneously, and memory plasma cells are among the CD19-negative plasma cell populations (33–35). Additionally, B cell activating factor (BAFF) cytokine, a member of the TNF superfamily, plays a crucial role in promoting the survival and function of B cells and memory plasma cells (36, 37). BAFF can bind to BAFF-receptor (BAFFR), Transmembrane Activator and CAML-interactor (TACI) and B-Cell Maturation Antigen (BCMA) and these three receptors have distinctive roles in regulating B cells function (37, 38). Overexpression of BAFF leads to the development of autoreactive B cells and displays autoimmune-like symptoms in mice, highlighting the significance of dysregulated BAFF expression in autoimmunity (39). Moreover, autoimmune diseases have been associated with sustained high levels of BAFF, making inhibiting BAFF signaling a promising therapeutic approach (40–43). Further, BCMA has shown to be essential for survival of plasma cells and memory plasma cells and increased expression of BCMA has been observed in SLE patients (44, 45). Therefore, targeting both B and memory plasma cells may be more effective, leading to the complete removal of autoantibodies. By combining various types of CARs with CD19, such as BAFF, BCMA or BAFFR, the effectiveness of CAR-T cells could be improved. This can be achieved by engineering two pools of T cells, each expressing different CARs, or by incorporating multiple antigen recognition domains within a single CAR construct, called a compound CAR (cCAR) (46). Recently, an early phase 1 clinical trial has started to evaluate the effectiveness of CD19-BAFF CAR-T cell therapy for autoimmune diseases (NCT06279923). Nevertheless, several clinical trials have started investigating the safety and efficacy of CD19-BCMA CAR-T cell infusion in various autoimmune conditions (Table 1).

**Table 1:** Clinical trials using BCMA-CD19-CAR T therapy for autoimmune diseases.

Condition	NTC number
Refractory systemic lupus erythematosus	05030779
Refractory systemic lupus erythematosus	05846347
Refractory systemic lupus erythematosus	05858684
Relapsed/refractory systemic lupus erythematosus	05474885
Refractory scleroderma	05085444
Refractory immune nephritis	05085418
Refractory immune nephritis	05085418
Refractory Sjogren’s syndrome	05085431
Refractory POEMS syndrome, amyloidosis, autoimmune hemolytic anemia, vasculitis	05263817

## 4 Chimeric autoantibody receptor engineering in autoimmune diseases

The use of chimeric autoantibody receptor (CAAR) T cells to precisely target autoantigen-specific B cell subpopulations and overcome complete B cell depletion is an emerging area of research. For autoimmune diseases caused by specific autoantibodies produced by individual B cell clones, these therapeutic T cells can be genetically engineered with a CAR targeting specific antigen of autoantibodies on autoreactive cells to suppress or modulate the immune response without affecting healthy tissues. CAAR-T provides a more targeted and personalized approach compared to traditional immunosuppressive therapies. Similar to CD19-specific CAR-T cells, CAAR-Ts function analogously by specifically targeting autoantigens, leading to the destruction of pathological immune cells. Additionally, natural killer (NK) cells expressing CAAR can also specifically remove pathological B cells *in vitro* and could be potentially investigated in future clinical trials (47, 48). Preclinical findings have shown the encouraging potential of CAAR-T cells in treating several autoimmune conditions (48–51).

Namely, NMDAR encephalitis is the most common autoimmune encephalitis, leading to psychosis, seizures, and autonomic dysfunction caused by anti-NMDA receptor (NMDAR) autoantibodies. Preclinical research demonstrated the efficacy of NMDAR-specific CAAR-T cells in depletion and sustained reduction of autoantibody levels without off-target toxicity in a mouse model, suggesting phase I/II trials in the treatment of NMDAR encephalitis with NMDAR-CAAR T cells (50). Further, Pemphigus vulgaris (PV) is an autoimmune disease caused by autoantibodies to keratinocyte adhesion protein Dsg3. Eliminating anti-Dsg3 memory B cells could cure PV without general immunosuppression risks. An engineered chimeric autoantibody receptor (CAAR) that targets autoantigen Dsg3 has been shown to guide T cells to target and successfully eliminate autoreactive B cells *in vivo* in a humanized mouse model. CAAR-Ts demonstrated clear therapeutic potential for antibody-mediated autoimmune diseases without off-target toxicity (51). Two CAAR-T cell therapies for treating human autoimmune diseases have entered clinical trials. A phase I open-label study has begun to determine the maximum tolerated dose, infusion schedule, and safety of Dsg3-CAAR-T cells in patients with mucosal-dominant PV (NCT04422912). Also, another phase 1 study has begun evaluating the safety of MuSK-CAAR-T cell therapy for patients with Muscle-specific tyrosine kinase (MuSK) myasthenia gravis (NCT05451212). Moreover, various combinations of CAARs could be used to enhance effectiveness in conditions where pathogenic autoantibodies target multiple autoantigens. Nevertheless, further studies are needed to demonstrate the long-term safety and efficacy of CAAR-T cells.

## 5 Engineered Tregs (CAR-Tregs) in autoimmune diseases

Regulatory T lymphocytes (Tregs) are small heterogenous subpopulation of T lymphocytes, that play a vital role in maintaining immunological balance. Tregs suppress the immune response by limiting the ability of antigen-presenting cells to initiate an adaptive immune response, inducing apoptosis of effector T cells, disrupting metabolic pathways, and releasing anti-inflammatory cytokines (52). Dysregulation of these processes can lead to Treg dysfunction, which can also manifest in activation defects. This creates an imbalance in the ratio between resting and activated Tregs, contributing to autoimmune processes (53, 54). Moreover, Treg levels or functional alterations are associated with many autoimmune disorders, and decreased Treg frequencies have been identified in several autoimmune diseases, which may be linked to disease severity (52, 54–57). Altering the low frequency or dysfunction of Tregs is considered a novel approach to treating autoimmune disorders, with the primary goal of decreasing inflammation, facilitating tissue repair and restoring immune tolerance. Phase 1 clinical trials have indicated that infusion of autologous *ex vivo*-expanded Tregs is safe and well tolerated, with no significant adverse events (58). Antigen-specific Tregs have demonstrated greater efficacy and reduced risk of general immunosuppression compared to polyclonal Tregs in preclinical tests, indicating a potential therapeutic strategy for the future (59, 60). Several autoimmune diseases could benefit from antigen-specific CAR-Tregs, as described below.

Loss of Tregs has been identified as an important factor in the development of SLE (61). Moreover, Tregs in SLE patients have a reduced frequency and suppressive activity as well as an increased apoptosis rate compared to normal controls, suggesting new treatment options focused on Treg function (56). Overactive T cells in SLE can contribute to impaired immunological regulation, increased production of inflammatory cytokines, and an increase in effector T-cell phenotypes. IL-2 is a crucial factor for the expansion of Treg cells, and epigenetic silencing of the IL-2 gene in T cells leads to decreased production of IL-2, contributing to reduced Tregs and a secondary immune deficiency (62–64). Engineered anti-CD19 CAR-Tregs may improve their immune suppressive capabilities, block B cell proliferation, and restore the immune system’s normal composition in inflamed organs in a humanized mouse model of SLE (65).

Further, Tregs are essential in regulating CNS autoimmunity in several experimental autoimmune encephalomyelitis (EAE) models by limiting autoimmune inflammation through controlling cytokine secretion and modulating T effector cell proliferation and migration. Loss of Tregs worsens EAE severity, followed by increased pro-inflammatory cytokine production and proliferation of effector T cells, indicating that modulation of Tregs function could be an effective treatment approach (66). Animal models of EAE showed that CAR-Tregs could prevent, improve, and protect against the disease, effectively reducing or eliminating symptoms (67, 68). Furthermore, CAR-Tregs showed effectiveness in suppressing the manifestations of colitis in mouse models, indicating the viability of a CAR-Treg-based approach (69). Additionally, a study in mice examined antigen-specific CAR-Tregs for treating vitiligo, where GD3-reactive CAR-Tregs were generated. The results indicate that restoring peripheral Tregs can protect against skin depigmentation and that CAR-Tregs lead to increased secretion of IL-10, limit cytotoxicity toward melanocytes and delay depigmentation. This implies that antigen-specific CAR-Treg cells may have a positive effect on the control of long-term progressive depigmentation (70).

Type 1 diabetes mellitus (T1DM) is an autoimmune condition that attacks and destroys *β* cells in the pancreas. Although the frequency of Tregs in T1DM is normal, they exhibit an activation defect characterized by an increase in resting Tregs and a decrease in activated Tregs. A lower number of activated Tregs has been linked to a more severe clinical course, indicating its clinical significance (54). Therefore, delivering, redirecting, and activating T regulatory cells to target *β* cells could modulate the immune system and have therapeutic effects. Insulin-specific CAR-Tregs obtained from CD4+ effector T cells have shown promising results in a mouse model of type 1 diabetes. These CAR-Tregs had a normal Treg phenotype, suppressive function, and stability. Although they could not prevent diabetes in mice, they have demonstrated clear therapeutic potential with minimal off-target toxicity (71). Further, a recent study aimed to evaluate the potential of using HPi2 targeting CAR-Tregs to restore immune balance and improve disease in T1D *in vitro*. HPi2 was hypothesized to target all human endocrine pancreatic islet cell subtypes, and this approach was chosen since autoimmune processes typically destroy most endogenous *β* cells. However, the study found that anti-HPi2-driven CAR-Tregs were unsuccessful due to their non-specificity for human pancreatic islets and high expression on CD4+ T cells, suggesting future studies should explore alternative islet-specific targets (72).

Rheumatoid arthritis (RA) is a common chronic autoimmune disease causing irreversible joint and bone damage. Tregs have been found to have impaired function or number in RA patients and thus could be used as a promising therapeutic target (73–76). A phase I clinical trial is underway to assess the safety and efficacy of an autologous CAR-Treg cell-based therapy for treating RA (NCT06201416). This therapy specifically targets citrullinated proteins accumulated in the inflamed tissue associated with the disease to reduce inflammation and restore immune system tolerance.

Altogether, additional research is needed to investigate the effectiveness of CAR-Tregs, identify disease-specific targets, improve the manufacturing process by defining suitable sources for Treg isolation, and improve marker selection and proliferation capacity (77).

## 6 Potential adverse effects and future perspectives

CAR-T cell therapy has shown promise in treating hematological cancers, but it can lead to several potential side effects, including cytokine release syndrome (CRS) and immune effector cell-associated neurotoxicity syndrome (ICANS). CRS is the most common and potentially life-threatening inflammatory response caused by the rapid activation and expansion of CAR-T cells. This can result in the excessive release of cytokines, leading to symptoms such as high fever, hypotension, and in rare cases multi-organ failure. ICANS is linked to CRS but presents distinct symptoms like confusion, seizures, and cerebral edema. CRS often precedes neurotoxic events, suggesting a temporal and mechanistic link (78–80). A recent systematic review and meta-analysis involving 7,604 patients looked into the rate of non-relapse mortality (deaths not caused by cancer progression) in various CAR T-cell trials for lymphoma and multiple myeloma. Non-relapse mortality rates varied from 1 to 10% across trials, with only a minority associated with the most common side effect, CRS. Additionally, 50.9% of non-relapse mortalities were due to infections, underscoring the critical importance of addressing infectious complications after CAR T cell therapy (81).

So far, CAR-T cell therapy have shown encouraging results, with demonstrated feasibility, tolerability, and efficacy in treating autoimmune disorders. However, more long-term assessments are required before they are accepted in wide clinical applications. The FDA recently reported T-cell malignancies in patients treated with BCMA-or CD19-directed autologous CAR T-cell therapies. Secondary malignancy risk is a concern for all approved products in this category. While the advantages of these products still surpass the possible drawbacks of their authorized use, the FDA is examining the potential for severe consequences such as hospitalization and death and is considering regulatory measures (82). Therefore, safety for autoimmune trials must be at a higher rate. The choice of an adequate strategy for cellular engineering to treat autoimmune diseases depends on the pathogenesis underlying the disease, its severity and length, and associated conditions. Additionally, due to the complex and heterogeneous nature of autoimmune diseases, other targets, as well as the efficacy and safety of multitargeting, should be further investigated.

Furthermore, the duration of optimal CAR-T cell activity in the treatment of autoimmune diseases is a subject of debate. In cancer treatment, long-term persistence of CAR T cells is beneficial for maintaining continuous immune monitoring. However, in autoimmune diseases, prolonged CAR T cell activity may result in immunosuppression or organ damage due to excessive elimination of normal immune cells. Therefore, it may be preferable to have limited or controlled expression of CAR T cells in the treatment of autoimmune diseases to minimize potential long-term toxicities. Therefore, future strategies should implement molecular switches or shorter half-life constructs CARs that express transiently or degrade after a specific period to reduce long-term immune suppression. The introduction of molecular switches can help manage immune-mediated toxicities, such as CRS and ICANS. Suicide switches provide a way to rapidly destroy CAR-T cells in cases of severe toxicity, ensuring safety. On/off switches offer external control by activating CAR expression only in the presence of a specific drug, allowing for precise titration of therapy based on patient response. Additionally, logic gate systems like AND and NOT gates improve targeting specificity, activating CAR-T cells only when certain conditions are met, thus minimizing damage to healthy tissues. These innovations enhance safety and efficacy, making CAR-T therapy more adaptable for complex diseases like autoimmunity (83–86).

Shorter half-life constructs CARs such as messenger RNA (mRNA)-based CAR T cells offer an alternative strategy to achieve transient and restricted expression of CARs, providing for more controlled treatment. Moreover, this also allows for *in vivo* reprogramming of T cells, favoring this approach due to faster production and lower cost (87, 88).

Manufacturing-related issues, including variability in cell quality and production delays, can also affect treatment outcomes. Some drawbacks of CAR-T therapy are the long production time (around 30 days) and high cost (up to half a million dollars) due to the autologous manufacturing process. Thus, alternative strategies focused on resolving these issues are under investigation. Decentralized production or on-site production using a fully automated closed system could significantly shorten the manufacturing process and lower the therapy cost (89, 90).

Recently, the European Society for Blood and Marrow Transplantation (EBMT) and the International Society for Cell and Gene Therapy (ISCT) published expert-based consensus and recommendations on using cellular therapies, including CAR-T, for treating severe and refractory autoimmune diseases. The guidelines emphasize referrals to expert centers with inter-disciplinary interaction, including hematological and autoimmune diseases specialist experience, as well as assessment of the effectiveness and tolerability of the therapies, long-term outcomes, and safety monitoring (91).

In conclusion, CAR-T cell therapy offers significant advantages over conventional treatments for autoimmune diseases by addressing the root cause of the condition: the autoreactive immune cells. Unlike traditional therapies, which broadly suppress the immune system, CAR-engineered T cell therapy holds the potential to specifically target and eliminate the pathogenic immune cells driving the autoimmune response. This precise approach helps preserve overall immune function, reducing the risk of infections and other side effects. Additionally, CAR-T cells can be engineered with molecular switches that allow real-time control over their activity, enhancing both safety and efficacy. This strategy offers the potential for long-term remission by directly targeting the underlying drivers of autoimmunity, rather than merely managing symptoms. Undoubtedly, CAR-T cell therapy stands as a compelling therapeutic approach for various autoimmune diseases, although there are still unresolved challenges that need to be addressed for widespread clinical use.
